# The Impact of Adjustment for Socioeconomic Status on Comparisons of Cancer Incidence between Two European Countries

**DOI:** 10.1155/2013/612514

**Published:** 2013-12-22

**Authors:** David W. Donnelly, Avril Hegarty, Linda Sharp, Anne-Elie Carsin, Sandra Deady, Neil McCluskey, Harry Comber, Anna Gavin

**Affiliations:** ^1^Northern Ireland Cancer Registry, Centre for Public Health, Mulhouse Building, Grosvenor Road, Belfast BT12 6DP, UK; ^2^MACSI, Department of Mathematics and Statistics, University of Limerick, Limerick, Ireland; ^3^National Cancer Registry, Building 6800, Cork Airport Business Park, Kinsale Road, Cork, Ireland; ^4^Centre for Research in Environmental Epidemiology (CREAL), Barcelona, Spain; ^5^CIBER Epidemiología y Salud Pública (CIBERESP), Barcelona, Spain

## Abstract

*Background.* Cancer incidence rates vary considerably between countries and by socioeconomic status (SES). We investigate the impact of SES upon the relative cancer risk in two neighbouring countries. *Methods.* Data on 229,824 cases for 16 cancers diagnosed in 1995–2007 were extracted from the cancer registries in Northern Ireland (NI) and Republic of Ireland (RoI). Cancers in the two countries were compared using incidence rate ratios (IRRs) adjusted for age and age plus area-based SES. *Results.* Adjusting for SES in addition to age had a considerable impact on NI/RoI comparisons for cancers strongly related to SES. Before SES adjustment, lung cancer incidence rates were 11% higher for males and 7% higher for females in NI, while after adjustment, the IRR was not statistically significant. Cervical cancer rates were lower in NI than in RoI after adjustment for age (IRR: 0.90 (0.84–0.97)), with this difference increasing after adjustment for SES (IRR: 0.85 (0.79–0.92)). For cancers with a weak or nonexistent relationship to SES, adjustment for SES made little difference to the IRR. *Conclusion.* Socioeconomic factors explain some international variations but also obscure other crucial differences; thus, adjustment for these factors should not become part of international comparisons.

## 1. Introduction

International comparisons of cancer incidence highlight considerable differences in incidence rates between various countries [1]. The incidence rates in these studies are routinely age-adjusted due to the relationship between cancer and age and the variation between countries in their demographic makeup. It has been well established that socioeconomic status (SES) also influences the incidence rate of many types of cancer [2]. However, it is not usual to take into account the relationship between cancer and SES in international comparisons.

The Republic of Ireland (RoI) and Northern Ireland (NI) are the only two countries on the island of Ireland, although NI is one of the constituent countries making up the United Kingdom. Recent studies have shown differences between the two countries in incidence rates for lung, bladder, brain, prostate, cervical, uterine and male colorectal cancer, leukaemia, and female melanoma [3]. This is despite the proximity of the two countries and their similar demographics and proportion of different ethnic groups [4, 5]. Additionally in the 2001 NI [4] and 2002 RoI censuses [5] 40% of the NI 16–74-year-old population was economically inactive compared to 34% in RoI, while of the economically active population 7% in NI was unemployed compared to 6% in RoI. However the cancer services of the two countries are different, with the RoI system a mixture of public and private provision and the NI system mostly public.

Given the relationship between some cancers and SES [2], the differences in cancer incidence rates between NI and RoI may thus be partially due to the different socioeconomic situations in each country. This paper investigates the extent to which observed differences in cancer incidence between these two neighbouring countries are explained by these socioeconomic variations.

## 2. Methods

Data on 229,824 cases of the 16 most common cancers (excluding non-melanoma skin cancer) diagnosed in 1995–2007 on the island of Ireland were extracted from the cancer registries in NI and RoI. Multiple primary cancers were excluded in the calculation of incidence figures based upon the rules published by the International Agency for Research on Cancer [6]. Cases were allocated to cancer sites based upon their ICD10 codes [7] (Table 1).

Geocoding of cancer cases (i.e., assigning cases to small geographic units) is routinely performed by each cancer registry. In NI, cases are assigned to electoral wards using a postcode-to-electoral ward lookup file known as the Central Postcode Directory (CPD) [8]. In RoI, addresses are coded to electoral divisions (ED) by means of matching to other data sources such as the GeoDirectory database [9] which provides a list of official postal addresses and location details for every property in the country. Some registrations in RoI and NI could not be assigned to any ED/ward (3.6% in RoI and 2.7% in NI). For these registrations, a fraction of the cases of each cancer type was allocated in proportion to each ED (RoI) or ward (NI) weighted by population.

In NI population, estimates for each year are available by sex and age at district council level [10]. Annual estimates for the 582 wards were derived from these estimates using the 2001 census [4] as the basis for the splits by ward. Three censuses were carried out in RoI during the study period, in 1996, 2002, and 2006 [5, 11]. These censuses provided population data, broken down by sex and age, for each ED. For confidentiality reasons and changes in boundaries over time, some EDs were merged resulting in 3,355 EDs. Official estimates of the total RoI population split by sex and age were available for each year from 1995 to 2007 [12]. Annual estimates for the EDs were derived using linear interpolation from the appropriate census constrained by the total annual population estimates. Over this period, the electoral wards in NI had an average population of 2,913 (ranging from 784 to 9,654), while the average ED population in RoI was 1,161 (ranging from 62 to 33,983).

While a wide range of area-based socioeconomic measures were available from the population censuses in NI and RoI, the majority of these, particularly those relating to occupation and social class, use different definitions in NI and RoI and are not directly comparable [4, 5, 11]. The most directly comparable measure was unemployment, as both countries use the definition from the International Labour Office (ILO) [13], namely, the proportion of the economically active population aged 16–74 who were unemployed. This definition has also remained consistent during the period covered by this study.

Wards and EDs were ranked according to the increasing levels of unemployment and were divided into population quintiles for the entire island based on the population data from the 2001 NI and 2002 RoI censuses [4, 5]. Thus, the 20% of the all-Ireland population resident in areas with the lowest unemployment was assigned to socioeconomic status (SES) quintile 1 (highest SES), while the 20% resident in areas with the highest unemployment was assigned to SES quintile 5 (lowest SES).

A count of the observed number of cancer cases by type and sex were generated for each ward/ED, while the expected number of cases in these areas was calculated by applying the all-Ireland incidence rates for each age group to the population counts in the equivalent age group. Relating these counts to the ward/ED characteristics was done using negative binomial regression to adjust more fully for overdispersion in the data [14]. The analysis was conducted twice, firstly to generate a cancer incidence rate ratio (IRR) for NI relative to RoI (which was used as the reference category) adjusted for age only and secondly to generate an IRR adjusted for both age and socioeconomic status.

## 3. Results

The average population on the island of Ireland during 1995–2007 was 5,590,087, with 3,894,549 (70%) residents in RoI, and the remainder resident in NI. While overall 20% of the population of the island was resident in each SES quintile, 30% of the NI population lived in the areas of lowest SES (quintile 5), compared to 16% of the RoI population (see Table 1 in Supplementary Material available online at http://dx.doi.org/10.1155/2013/612514).

A strong, positive relationship (IRR comparing SES quintiles 1 and 5 (IRR_Q1–Q5_) >1.2) between cancer and socioeconomic status was found for lung, head and neck, stomach, female bladder, and cervical cancer, while a strong, negative relationship (IRR_Q1–Q5_ < 0.8) was found for melanoma. Weak, positive (1.0 < IRR_Q1–Q5_ < 1.2) but statistically significant relationships were present for male oesophageal, colorectal, and bladder cancer and for female kidney cancer, while weak, negative (0.8 < IRR_Q1–Q5_ < 1.0) but statistically significant relationships were present for breast (female only) and prostate cancers (Supplementary Table  2).

**Figure 1:**
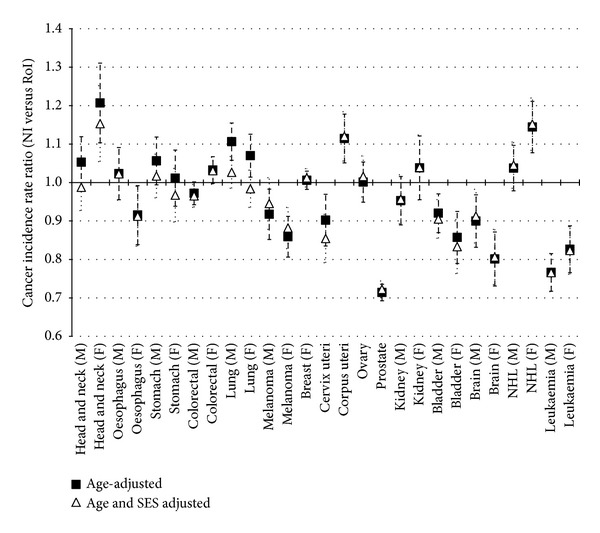
Cancer incidence rate ratios with 95% confidence intervals—Northern Ireland compared to Republic of Ireland adjusted for (a) age and (b) age and socioeconomic status.

After adjusting for age, only the risk of lung cancer among males and females head and neck cancer, cancer of the corpus uteri, and non-Hodgkin's lymphoma among females was significantly higher in NI than in RoI. Conversely the risk of melanoma, bladder cancer, brain (including central nervous system) cancer and leukaemia among males and females, prostate cancer among males, cancer of the cervix uteri among females, and oesophageal cancer among females was significantly lower in NI than in RoI. There was no significant difference between the two countries for breast, colorectal, stomach, kidney, ovarian, male head and neck, male oesophageal cancers or for male non-Hodgkin's lymphoma (Figure 1, Supplementary Table  3).

Adjusting for SES in addition to age had a considerable impact on the cancer incidence rate ratio between NI and RoI for those cancers with a strong positive relationship with SES (lung, stomach, head and neck, cervix, and female bladder). Before adjustment for SES lung cancer was 11% higher for males and 7% higher for females in NI than in RoI, while after adjustment there was no longer a significant difference between the two countries. The cancer IRR between NI and RoI changed by 4% for male and female stomach cancer and by 6% for male head and neck cancer after adjustment for SES, although there was no significant difference between the two countries before or after SES adjustment. Female head and neck cancer was 21% higher in NI than in RoI, a ratio which was reduced to 15% after adjustment for SES. Cervical cancer however was lower in NI than in RoI after adjustment for age only (IRR: 0.90 (0.84–0.97)). This difference increased by a further 5% after adjustment for SES (IRR: 0.85 (0.79–0.92)). Similarly, the IRR for female bladder cancer changed from 0.86 (0.79–0.93) to 0.83 (0.76–0.91) as a result of SES adjustment (Figure 1, Supplementary Table  3).

Only melanoma had a strong negative relationship to SES. The melanoma IRR comparing NI and RoI changed marginally when adjusted for SES, rising from 0.92 (0.85–0.99) for males and 0.86 (0.81–0.92) for females when adjusted for age only to 0.95 (0.88–1.02) for males and 0.88 (0.83–0.94) for females when adjusted for age and SES (Figure 1, Supplementary Table  3).

The remaining cancers either had no relationship to SES or had a weak relationship. For these cancers, the adjustment for SES made little difference to the cancer incidence rate ratio comparing NI to RoI (Figure 1, Supplementary Table  3).

## 4. Discussion

The data utilized in this study comes from the two cancer registries in Ireland, both of which follow the same international conventions with respect to registration and coding, with data from each having been independently verified as being of a high quality [15]. However, this study is limited in that it uses area-based unemployment as a proxy for the socioeconomic status of individual cancer patients. This has been necessitated by the lack of comparable small area socioeconomic measures between RoI and NI and the lack of individual level SES data gathered by the two cancer registries, a problem which exists in most international cancer registries. Encouragingly, previous studies have illustrated that the use of area-based employment at an electoral ward level in NI gives a similar relationship (both in magnitude and direction) as when income is used [16], while linkage of cancer registry data to the census in the US illustrated that area-based measures give similar results to individual level unemployment status for the top four cancers [17].

Despite the use of area-based unemployment as a proxy for socioeconomic status, the relationships between SES and cancer observed in this study agree well with previous studies conducted in various countries using both area-based and individual level SES data [2, 3, 17–19]. Lung, head and neck, cervical, female bladder, and stomach cancer incidence rates were all considerably higher among those with low SES, while melanoma incidence rates were considerably higher among those with high SES. Weak relationships with high SES were present for breast and prostate cancers, while weak relationships with low SES were also found for oesophageal, colorectal, and kidney cancers, although the relationship was not always present for both sexes. The relationship for bladder and kidney cancers is not a globally established phenomenon with only some countries demonstrating a relationship between SES and these cancers [2]; however, this relationship has been previously identified in NI, and RoI [3] and in the UK [19]. Thus, we would suggest that in international comparisons, in the absence of individual level SES indicators that are comparable between countries, the use of area-based unemployment is a reasonable substitute.

During the time of the study, the overall percentage of people unemployed in NI was only 1% higher than in RoI; however, we found that considerably more people were living in areas of high unemployment in NI, while a similar proportion of the populations of each country were living in areas of low unemployment. This is likely due to a higher level of correlation between economic deprivation and urban residence in NI than in RoI, with unemployed people in NI living in closer proximity to those living on benefits and those with a low income.

Consequently for cancers with a strong relationship to low SES and higher age-adjusted incidence rates in NI than in RoI (lung, head and neck, and stomach cancer), the relative differences in rates between the two countries decreased after adjustment for SES. In contrast, when the age-adjusted incidence rates were higher in RoI than in NI (female bladder and cervical cancer), the relative difference in rates increased after SES adjustment. The only cancer with a strong relationship to high SES was melanoma which was higher in RoI than in NI when adjusted for age only, and the relative difference in rates decreased when adjustment was also made for SES.

Incidence of lung cancer is strongly related to tobacco use [20]. With smoking levels higher among lower social classes [21], the adjustment for SES is likely to be indirectly adjusting the RoI/NI incidence rate ratio for smoking prevalence. Adjusting for SES in stomach, head and neck, and bladder cancer comparisons is likely to work in a similar way; however, in addition to smoking [20] other risk factors, such as alcohol consumption for various head and neck cancers [22] and poor diet for stomach cancer [23], also influence risk of developing these cancers. The Survey of Lifestyle, Attitudes and Nutrition [21] and NI Health and Social Well Being Survey [24] in RoI and NI, respectively, report that while the proportion of people who drink alcohol was lower among lower social classes, among those who do drink the frequency and volume of alcohol consumed was higher among lower social classes. In addition, the proportion taking the recommended level of physical activity and consuming the recommended amount of fruit and vegetables was higher among higher social classes.

Cervical cancer is primarily caused by the human papillomavirus (HPV) [25], which has been shown in some studies to be more prevalent in socioeconomically deprived areas [26]. Given the greater proportion of the NI population resident in areas of low SES, we would expect NI to have higher rates of cervical cancer than RoI. However, the increased risk in NI is compensated for by the long-term presence of a national organised population-based screening program geared at reducing cervical cancer incidence rates. Such a program was only introduced in RoI after the period covered in this study. Consequently rates of cervical cancer in RoI were higher than those in NI, and this difference increased once adjustment was made for SES. Thus, in this particular instance, the higher levels of socioeconomic deprivation in NI and the associated higher cervical cancer risk, partially, conceal the benefits of the screening program in NI with regard to reducing cervical cancer incidence compared to countries, such as RoI, which have fewer areas of high deprivation yet do not have a screening program.

Melanoma is primarily related to UV exposure [27], with the variation in melanoma rates by SES related to but not completely explained by variation in UV exposure by SES [28]. Adjustment for SES reduces, but does not eliminate, the difference in melanoma rates between NI and RoI, which remain significantly higher in RoI. This is likely due to its more southerly location and higher proportion of coastline resulting in higher sunshine levels [29] and increased UV exposure.

Of the remaining cancers, none showed any major change in relative rates between the two countries as a result of adjustment for SES, either due to the a weak or a nonexistent relationship to socioeconomic status. Thus, the explanations for the differences in rates of several cancers between NI and RoI, including prostate, brain, male bladder cancer, and leukaemia, must not be connected to variations in socioeconomic status.

## 5. Conclusion

Socioeconomic factors impact upon international comparisons of incidence for certain cancers. For four of the six cancers with a strong relationship to SES (lung cancer, head and neck cancer, stomach cancer, and melanoma), the difference in incidence rates between RoI and NI was either eliminated or considerably reduced by adjustment for SES, while for two cancers (cervical and female bladder) the difference was increased. The changes in relative rate were likely to be a result of the relationship between SES and exposure to risk factors and—for cervical cancer—availability of organised screening. Consequently, we do not recommend that international comparisons are routinely adjusted for SES as this may mask underlying risk factors. However, as evidenced by the elimination of lung cancer differences after SES adjustment such adjustment may be useful in identifying why such differences exist. In conclusion, therefore, adjustment for SES can thus assist in elucidating international differences, but it should not become a standard part of international comparisons.

## Supplementary Material

The supplementary tables provide further background data on the analysis conducted and the results produced. Table 1 provides population data and the number of areas (wards and EDs) included in each socio-economic status category. Table 2 includes cancer incidence rate ratios (with 95% confidence intervals) for socio-economic quintiles adjusted for age. This table thus describes the relationship between cancer incidence and socio-economic status in Ireland during the period of the study. Table 3 presents cancer incidence rate ratios (with 95% confidence intervals) for Northern Ireland compared to Republic of Ireland adjusted for age and age plus socio-economic status. This table includes the main results from the study which form the basis for figure 1 included in the article.

## Figures and Tables

**Table 1:** Numbers of cancers diagnosed during 1995–2007 in Republic of Ireland and Northern Ireland, by sex.

Cancer site	ICD10 code	Republic of Ireland		Northern Ireland	
		Males	Females	Males	Females
Head and neck	C01–C14, C30–C32	3,820	1,371	1,872	840
Oesophagus	C15	2,627	1,592	1,280	778
Stomach	C16	3,818	2,353	1,930	1,263
Colorectal	C18–C21	14,485	11,041	6,720	5,951
Lung	C34	13,672	8,437	7,159	4,568
Melanoma	C43	2,611	3,871	1,091	1,596
Breast	C50	181	25,876	73	12,669
Cervix uteri	C53	—	2,665	—	1,093
Corpus uteri	C54	—	3,355	—	1,882
Ovary	C56	—	4,149	—	2,073
Prostate	C61	24,704	—	8,440	—
Kidney	C64–C65	2,785	1,603	1,247	847
Bladder	C67	4,309	1,730	1,917	783
Brain	C70–C72	2,161	1,630	880	636
Non-Hodgkin's lymphoma	C82–C85	3,441	2,917	1,653	1,688
Leukaemia	C91–C95	3,331	2,235	1,196	929
