# Supplementary material for: The Impact of Adjustment for Socioeconomic Status on Comparisons of Cancer Incidence between Two European Countries
Source: J Cancer Epidemiol. 2013 Dec 22;2013:612514. doi: 10.1155/2013/612514 (PMC3881585; doi:10.1155/2013/612514)
Supplement: Supplementary file 1 — The supplementary tables provide further background data on the analysis conducted and the results produced. Table 1 provides population data and the number of areas (wards and EDs) included in each socio-economic status category. Table 2 includes cancer incidence rate ratios (with 95% confidence intervals) for socio-economic quintiles adjusted for age. This table thus describes the relationship between cancer incidence and socio-economic status in Ireland during the period of the study. Table 3 presents cancer incidence rate ratios (with 95% confidence intervals) for Northern Ireland compared to Republic of Ireland adjusted for age and age plus socio-economic status. This table includes the main results from the study which form the basis for figure 1 included in the article. [file 612514.f1.pdf]

Supplementary table 1: Population and number of areas (wards and EDs) included in each SES category

	Republic of Ireland			Northern Ireland			Ireland		
	**Number of areas**	**Population***	**% of total population**	**Number of areas**	**Population***	**% of total population**	**Number of areas**	**Population***	**% of total population**
**Highest SES (Q1)**	967	754,815	19%	114	358,345	21%	1,081	1,113,160	20%
**Quintile 2**	695	894,496	23%	78	225,691	13%	773	1,120,187	20%
**Quintile 3**	672	831,031	21%	104	283,577	17%	776	1,114,608	20%
**Quintile 4**	593	803,616	21%	121	320,098	19%	714	1,123,713	20%
**Lowest SES (Q5)**	428	610,592	16%	165	507,827	30%	593	1,118,419	20%
**Total**	3,355	3,894,549		582	1,695,538		3,937	5,590,087	

** Annual average of combined 1995-2007 population.*

Supplementary table 2: Cancer incidence rate ratios (with 95% confidence intervals) for SES quintiles adjusted for age, all Ireland, 1995-2007*

Cancer site	Sex	Cancer incidence rate ratio				
		Quintile 1 (highest SES)	Quintile 2	Quintile 3	Quintile 4	Quintile 5 (lowest SES)
Head & neck	Male	1.00	1.03 (0.94,1.14)	**1.13 (1.02,1.24)**	**1.31 (1.19,1.43)**	**1.65 (1.51,1.81)**
	Female	1.00	1.09 (0.94,1.26)	1.07 (0.93,1.24)	**1.24 (1.08,1.43)**	**1.58 (1.38,1.81)**
Oesophagus	Male	1.00	**1.13 (1.02,1.26)**	**1.14 (1.02,1.26)**	**1.22 (1.10,1.35)**	**1.18 (1.06,1.31)**
	Female	1.00	0.99 (0.87,1.13)	0.99 (0.87,1.13)	1.01 (0.89,1.15)	1.00 (0.88,1.14)
Stomach	Male	1.00	**1.11 (1.01,1.22)**	**1.12 (1.02,1.23)**	**1.22 (1.11,1.33)**	**1.46 (1.34,1.60)**
	Female	1.00	1.06 (0.94,1.19)	1.06 (0.94,1.19)	**1.21 (1.08,1.35)**	**1.45 (1.30,1.62)**
Colorectal	Male	1.00	**1.07 (1.02,1.12)**	**1.08 (1.04,1.13)**	**1.10 (1.05,1.15)**	**1.13 (1.08,1.18)**
	Female	1.00	1.02 (0.97,1.08)	1.01 (0.96,1.06)	1.01 (0.96,1.06)	1.04 (0.99,1.09)
Lung	Male	1.00	**1.14 (1.07,1.21)**	**1.15 (1.08,1.22)**	**1.41 (1.33,1.49)**	**1.75 (1.65,1.85)**
	Female	1.00	**1.14 (1.06,1.23)**	**1.19 (1.10,1.28)**	**1.46 (1.36,1.57)**	**1.78 (1.66,1.91)**
Melanoma	Male	1.00	1.04 (0.95,1.15)	**0.88 (0.80,0.98)**	**0.84 (0.76,0.93)**	**0.73 (0.65,0.81)**
	Female	1.00	**0.91 (0.84,0.99)**	**0.82 (0.75,0.90)**	**0.82 (0.75,0.89)**	**0.67 (0.61,0.73)**
Breast	Female	1.00	1.00 (0.96,1.03)	**0.95 (0.92,0.99)**	**0.97 (0.93,1.00)**	**0.93 (0.90,0.97)**
Cervix Uteri	Female	1.00	1.12 (1.00,1.26)	**1.20 (1.07,1.34)**	**1.43 (1.28,1.60)**	**1.63 (1.46,1.82)**
Corpus Uteri	Female	1.00	1.01 (0.93,1.11)	1.03 (0.94,1.12)	1.03 (0.94,1.12)	1.01 (0.93,1.11)

**Ovary**	**Female**	1.00	1.04 (0.96,1.12)	**1.10 (1.01,1.19)**	1.07 (0.99,1.15)	0.96 (0.88,1.04)
**Prostate**	**Male**	1.00	1.04 (0.99,1.08)	0.97 (0.93,1.01)	**0.95 (0.91,1.00)**	**0.87 (0.83,0.91)**
**Kidney**	**Male**	1.00	1.04 (0.94,1.15)	1.01 (0.91,1.12)	**1.16 (1.05,1.27)**	1.01 (0.92,1.12)
	**Female**	1.00	**1.17 (1.03,1.33)**	1.10 (0.96,1.25)	1.11 (0.98,1.27)	**1.16 (1.02,1.32)**
**Bladder**	**Male**	1.00	1.05 (0.96,1.14)	1.02 (0.94,1.11)	**1.09 (1.01,1.18)**	**1.16 (1.07,1.26)**
	**Female**	1.00	1.04 (0.91,1.19)	1.05 (0.92,1.19)	1.07 (0.94,1.21)	**1.22 (1.08,1.39)**
**Brain**	**Male**	1.00	1.09 (0.97,1.21)	1.09 (0.97,1.22)	0.96 (0.86,1.08)	0.94 (0.85,1.07)
	**Female**	1.00	1.07 (0.94,1.22)	1.05 (0.93,1.20)	0.95 (0.83,1.08)	0.95 (0.84,1.09)
**Non-Hodgkin's Lymphoma**	**Male**	1.00	1.07 (0.98,1.16)	1.03 (0.95,1.13)	0.99 (0.91,1.08)	1.00 (0.92,1.09)
	**Female**	1.00	0.97 (0.89,1.06)	0.97 (0.89,1.07)	0.92 (0.84,1.01)	0.94 (0.86,1.03)
**Leukaemia**	**Male**	1.00	0.96 (0.88,1.05)	0.96 (0.88,1.06)	0.96 (0.88,1.05)	0.91 (0.83,1.00)
	**Female**	1.00	0.96 (0.86,1.08)	0.98 (0.88,1.09)	0.99 (0.88,1.10)	0.95 (0.85,1.06)

* IRRs shown in bold font are statistically significantly different from 1 ($p<0.05$)

Supplementary table 3: Cancer incidence rate ratios (with 95% confidence intervals) - Northern Ireland compared to Republic of Ireland adjusted for (a) age and (b) age and socio-economic status (SES)

Cancer site	Cancer incidence rate ratio (NI vs. RoI)			
	Male		Female	
	Age-adjusted	Age and SES adjusted	Age-adjusted	Age and SES adjusted
**Head & neck**	1.05 (0.99,1.12)	0.99 (0.93,1.05)	**1.21 (1.10,1.32)**	**1.15 (1.05,1.26)**
**Oesophagus**	1.02 (0.96,1.10)	1.02 (0.95,1.10)	**0.92 (0.84,1.00)**	**0.91 (0.83,1.00)**
**Stomach**	1.06 (0.99,1.12)	1.02 (0.96,1.08)	1.01 (0.94,1.09)	0.97 (0.90,1.04)
**Colorectal**	0.97 (0.94,1.00)	**0.97 (0.94,1.00)**	1.03 (1.00,1.07)	1.03 (1.00,1.07)
**Lung**	**1.11 (1.06,1.16)**	1.03 (0.99,1.07)	**1.07 (1.01,1.13)**	0.98 (0.94,1.03)
**Melanoma**	**0.92 (0.85,0.99)**	0.95 (0.88,1.02)	**0.86 (0.81,0.92)**	**0.88 (0.83,0.94)**
**Breast**			1.01 (0.98,1.03)	1.01 (0.99,1.04)
**Cervix Uteri**			**0.90 (0.84,0.97)**	**0.85 (0.79,0.92)**
**Corpus Uteri**			**1.11 (1.05,1.18)**	**1.12 (1.06,1.19)**
**Ovary**			1.00 (0.95,1.06)	1.02 (0.96,1.07)
**Prostate**	**0.71 (0.69,0.74)**	**0.72 (0.70,0.74)**		
**Kidney**	0.95 (0.89,1.02)	0.96 (0.89,1.02)	1.04 (0.96,1.13)	1.04 (0.95,1.13)
**Bladder**	**0.92 (0.87,0.97)**	**0.91 (0.85,0.96)**	**0.86 (0.79,0.93)**	**0.83 (0.76,0.91)**
**Brain**	**0.90 (0.83,0.97)**	**0.91 (0.84,0.99)**	**0.80 (0.73,0.88)**	**0.81 (0.73,0.89)**
**Non-Hodgkin’s Lymphoma**	1.04 (0.98,1.10)	1.05 (0.98,1.11)	**1.14 (1.08,1.22)**	**1.15 (1.08,1.22)**
**Leukaemia**	**0.77 (0.72,0.82)**	**0.77 (0.72,0.82)**	**0.83 (0.77,0.89)**	**0.82 (0.76,0.89)**

* IRRs shown in bold font are statistically significantly different from 1 ($p<0.05$)
